# 1325. Recalibrating Estimates of Pneumococcal Disease in Hospitalized Canadian adults from 2010 to 2017 with Use of an Extended Spectrum Serotype-specific Urine Antigen Detection

**DOI:** 10.1093/ofid/ofab466.1517

**Published:** 2021-12-04

**Authors:** Jason J LeBlanc, May ElSherif, Lingyun Ye, Donna MacKinnon-Cameron, Ardith Ambrose, Todd F Hatchette, Amanda L S Lang, Hayley D Gillis, Irene Martin, Walter Demczuk, Melissa K Andrew, Guy Boivin, William Bowie, Karen Green, Jennie Johnstone, Mark Loeb, Anne McCarthy, Allison McGeer, Makeda Semret, Sylvie Trottier, Louis Valiquette, Duncan Webster, Shelly McNeil

**Affiliations:** 1 Canadian Center for Vaccinology (CCfV), IWK Health Centre, Nova Scotia Health Authority (NSHA), and Dalhousie University, Halifax, Nova Scotia (NS), Halifax, NS, Canada; 2 Saskatchewan Health Authority, Regina, Saskatchewan, Canada; 3 National Microbiology Laboratory, Public Health Agency of Canada, Winnipeg, MB, Canada; 4 National Microbiology Laboratory (NML), Regina, MB, Canada; 5 Dalhousie University, Halifax, Nova Scotia, Canada; 6 Centre Hospitalier Universitaire de Québec, Québec, Québec, Quebec, QC, Canada; 7 Vancouver General Hospital, and University of British Columbia, Vancouver, BC, Vancouver, BC, Canada; 8 Mount Sinai Hospital, Toronto, ON, Toronto, Ontario, Canada; 9 Public Health Ontario and University of Toronto, Toronto, ON, Toronto, Ontario, Canada; 10 McMaster University, Hamilton, ON, Hamilton, Ontario, Canada; 11 Ottawa Hospital General Campus, Ottawa, ON, Ottawa, ON, Canada; 12 University of Toronto, Toronto, ON, Canada; 13 McGill University Health Centre, Montreal, QC, Montreal, QC, Canada; 14 Centre Hospitalier Universitaire de Québec, Québec, Québec (QC), Quebec, QC, Canada; 15 Université de Sherbrooke, Sherbrooke, Quebec, Canada, Sherbrooke, QC, Canada; 16 Saint John Regional Hospital, St. John, NB., Saint John, NB, Canada

## Abstract

**Background:**

Pneumococcal vaccine recommendations in Canada include both age- and risk-based guidance. This study aimed to describe the burden of vaccine-preventable pneumococcal community acquired pneumonia (pCAP) and invasive pneumococcal disease (IPD) by age in hospitalized adults.

**Methods:**

Active surveillance for all-cause CAP and IPD in hospitalized adults was performed from 2010 to 2017, including laboratory results, patient demographics, and outcomes. *Streptococcus pneumoniae* was detected using blood and sputum culture, or urine antigen detection (UAD). Serotype was assigned using Quellung reaction, PCR, or serotype-specific UADs spanning the 24 serotypes in PCV13 and PPV23 vaccines. Data were categorized by age (16-49, 50-64, 65+, and 50+ years) and over time.

**Results:**

11129 ACP cases and 216 cases of IPD (non-CAP) were identified. A laboratory test for *S. pneumoniae* was performed in 8912 of ACP cases, identifying 1264 (14.2%) as pCAP. Compared to non-pCAP, pCAP cases were more likely to be admitted to intensive care units and require mechanical ventilation. These serious outcomes, as well as mortality, were more prominent in bacteremic pCAP and IPD. Risk factors for death in pCAP included aged 75+ years, immune compromising conditions, and BMI < 18.5. When categorized by age, the proportion of individuals aged 65+ years for pCAP and IPD was 49.8% and 48.6%, and the 50-64 year age cohort represented 31.3% and 29.9%, respectively. The contributions of PCV13 and PPV23 serotypes remained relatively stable over time, and overall represented 57.6% and 90.9% for pCAP, and 35.0% and 72.0% for IPD, respectively.

**Conclusion:**

Seven years following infant PCV13 immunization programs in Canada, PCV13 and PPV23 serotypes in pCAP and IPD remained predominant causes of pneumococcal disease. Serious outcomes were particularly evident in adults 50+, suggesting pneumococcal vaccines should be encouraged in this age group.

**Disclosures:**

**Jason J. LeBlanc, PhD, FCCM, D[ABMM]**, **GSK** (Research Grant or Support)**Merck** (Grant/Research Support)**Pfizer** (Grant/Research Support) **Todd F Hatchette, MD**, **GSK** (Grant/Research Support)**Pfizer** (Grant/Research Support) **Melissa K. Andrew, MD, PhD**, **GSK** (Grant/Research Support)**Pfizer** (Grant/Research Support, Advisor or Review Panel member)**Sanofi** (Consultant, Grant/Research Support, Advisor or Review Panel member)**Seqirus** (Advisor or Review Panel member) **Allison McGeer, MSc,MD,FRCPC,FSHEA**, **GlaxoSmithKline** (Advisor or Review Panel member)**Merck** (Advisor or Review Panel member, Research Grant or Support)**Pfizer** (Grant/Research Support, Scientific Research Study Investigator, Advisor or Review Panel member) **Louis Valiquette, MD, M.Sc.**, **Cubist** (Consultant)**GSK** (Grant/Research Support)**Merck** (Consultant)**Optimer** (Consultant)**Pfizer** (Grant/Research Support) **Shelly McNeil, FRCPC, MD**, **GSK** (Grant/Research Support)**Pfizer** (Grant/Research Support)**Sinofi Pasteur** (Grant/Research Support)

